# Occupational Exposure to Bisphenol A (BPA): A Reality That Still Needs to Be Unveiled

**DOI:** 10.3390/toxics5030022

**Published:** 2017-09-13

**Authors:** Edna Ribeiro, Carina Ladeira, Susana Viegas

**Affiliations:** 1 GIAS-ESTeSL, Escola Superior de Tecnologia da Saúde de Lisboa, Instituto Politécnico de Lisboa, 1990-096 Lisbon, Portugal; carina.ladeira@estesl.ipl.pt (C.L.); susana.viegas@estesl.ipl.pt (S.V.); 2 Landscape, Environment, Agriculture and Food (LEAF), Instituto Superior de Agronomia, Universidade de Lisboa, Tapada da Ajuda, 1349-017 Lisboa, Portugal; 3 GIGM-ESTeSL, Escola Superior de Tecnologia da Saúde de Lisboa, Instituto Politécnico de Lisboa, 1990-096 Lisbon, Portugal; 4 Centro de Investigação em Saúde Pública, Escola Nacional de Saúde Pública, Universidade NOVA de Lisboa, 1600-560 Lisbon, Portugal

**Keywords:** bisphenol A (BPA), endocrine disruptor, genotoxicity, occupational exposure, exposure assessment, health effects

## Abstract

Bisphenol A (BPA), 2,2-bis(4-hydroxyphenyl) propane, is one of the most utilized industrial chemicals worldwide, with the ability to interfere with/or mimic estrogenic hormones with associated biological responses. Environmental human exposure to this endocrine disruptor, mostly through oral intake, is considered a generalized phenomenon, particularly in developed countries. However, in the context of occupational exposure, non-dietary exposure sources (e.g., air and contact) cannot be underestimated. Here, we performed a review of the literature on BPA occupational exposure and associated health effects. Relevantly, the authors only identified 19 studies from 2009 to 2017 that demonstrate that occupationally exposed individuals have significantly higher detected BPA levels than environmentally exposed populations and that the detection rate of serum BPA increases in relation to the time of exposure. However, only 12 studies performed in China have correlated potential health effects with detected BPA levels, and shown that BPA-exposed male workers are at greater risk of male sexual dysfunction across all domains of sexual function; also, endocrine disruption, alterations to epigenetic marks (DNA methylation) and epidemiological evidence have shown significant effects on the offspring of parents exposed to BPA during pregnancy. This overview raises awareness of the dramatic and consistent increase in the production and exposure of BPA and creates urgency to assess the actual exposure of workers to this xenoestrogen and to evaluate potential associated adverse health effects.

## 1. Introduction

Bisphenol A (BPA), 2,2-bis(4-hydroxyphenyl) propane, is a xenoestrogens and one and the most utilized industrial chemical in the world. BPA is utilized in the production of a variety of polymers such as polycarbonate plastics or epoxy resins, in the production of thermal paper, etc., and is therefore employed in the manufacture of a variety of indoor applications and consumer products. Currently, BPA is detected in a variety of items such as thermal printer paper, electronic equipment, water pipes, sports safety equipment, medical devices, tableware, plastic containers, in the lining of cans currently utilized for food and beverages, dental sealants among others [1]. Human exposure to BPA is acknowledged as a generalized phenomenon, at least in developed countries, since analysis of tissue and fluid samples revealed the presence of BPA in the majority of the individuals analyzed [2]. Ingestion of contaminated food is expected to contribute by more than 90% to the general BPA environmental exposure for all age groups [3]. However, BPA is also detected in waste water, drinking water, air and dust particles [4,5,6]. BPA has been found in 86% of house dust samples at levels ranging from 0.2 to 17.6 μg/g. In the urban outdoor environment, this compound has been detected in air samples at an average level of 0.51 ng/m^3^ associated with mild seasonal deviation in BPA levels, with higher levels from autumn to winter and lower levels from winter to Spring. Additionally, BPA has also been detected in air samples from workplace plastics (208 ng/m^3^) reviewed in [4]. Therefore, non-dietary human exposure routes such as air or contact cannot be underestimated [2,7,8], particularly in the context of occupational exposure [9,10]. 

Considering that the worldwide production and consumption of BPA is increasing dramatically, the number of individuals that are occupationally exposed to this compound is, consequently, increasing every year. Therefore, there is an urgent need to characterize the effective exposure of workers to BPA in different occupational settings which will allow the implementation of suitable preventive measures. 

## 2. Bisphenol A (BPA) State-Of-The-Art

BPA was synthesized by A. P. Dianin in 1891 and its estrogenic properties were hypothesized in the search for synthetic estrogens in the 1930s; however, when Diethylstilbestrol (DES) was found to be more effective, BPA was abandoned in pharmaceutical applications [11]. Since 1940, BPA has been extensively utilized in the production of a variety of consumer products [1]. In the 1960s, several studies emerged focused on the hypersensitivity and metabolism of BPA in different model systems [12,13,14]. Importantly, in 1993, Krishnan and co-workers accidentally discovered that BPA leaches from autoclaved polycarbonate flasks and showed, for the first time, its positive effect on the proliferation rate of the human cell line MCF-7 [15]. Since then, exhaustive research has been conducted on the effects of this endocrine disrupting chemical (EDC) on both animals and humans, reviewed in [11,16]. In 2001, the United States National Toxicology Program’s Report of the Endocrine Disruptors Low-Dose Peer Review recognized that there was credible but not conclusive evidence that low doses of BPA can cause effects on specific endpoints [17]. Since 2006, numerous scientific assessments have been conducted on BPA by the European Food Safety Authority (EFSA), repeatedly concluding that there is no concern for human health [18]. The metabolism and toxicokinetics of BPA have been studied in rodents [19,20], non-human primates [19,21] and humans [22,23]. Evaluation of the BPA excretion kinetic profile in a limited number of human volunteers subjected to a single oral dose of deuterated BPA revealed a rapid peak and a terminal half-life of less than 6 h [22,23]. These data have been inferred as indicating a rapid and complete BPA clearance in humans. However, large-scale biomonitoring data revealed that urine BPA levels do not decline rapidly with fasting time, suggesting a longer BPA clearance [24], which is also supported by the fact that, in the 2002 Volkel study, no significant removal of conjugated BPA was detected beyond 20 h of exposure [22]. 

Considering the results of worldwide studies, in 2011, the European Legislation abolished the use of BPA in the manufacture of baby bottles [25] and in 2012 EFSA decided to carry out a new risk assessment of BPA [18], which resulted in a temporary reduction of the established tolerable daily intake (TDI) (from 0.05 mg/kg to 4 μg/kg body weight/day). However, occupational exposure to BPA which results in significantly higher exposure levels than those resultant from environmental exposure, has, until now, been disregarded. 

## 3. BPA as an Endocrine Disruptor Chemical

Although BPA estrogenic activity has long been acknowledged, it has been considered a weak estrogen due to the fact that its binding affinity to classical estrogen receptors α and β (ERα and ERβ) is 10,000- and 1000-fold lower than that of endogenous estrogen estradiol (E2) for ERα and ERβ, respectively [26]. However, distinct studies have shown that BPA can, in fact, promote estrogen-like effects similar to or stronger than E2. BPA at a dose range of 0.1–1 nM was shown to be equally effective, or even more effective, than equimolar concentrations of E2 in suppressing adiponectin release from human adipose tissues [27]. In BG-1 ovarian cancer cells, both BPA and E2 induce cellular proliferation by promoting the interaction between ERα and IGF-1R signaling pathways [28]. BPA induction of alternative signaling pathways is also a plausible explanation for the paradigm of E2 and BPA equivalent effects. Interestingly, BPA binds to the orphan nuclear receptor estrogen-related receptor-γ (ERR-γ) [29] with 80–100 times higher affinity than for ERα or ERβ [30,31]. Also, activation of membrane-bound variants of ERs—particularly ERα, that act outside the nucleus—has been suggested as a possible BPA mode of action (reviewed in [32]). Additionally, cellular responses to BPA at low concentrations have been correlated with the trans-membrane estrogen receptor G protein-coupled estrogen receptor (GPR30 or GPER) for which BPA has relatively high affinity corresponding to 2.8% of that of E2 [30,33]. BPA induces rapid activation of the ERK signaling pathway through GPR30 in breast cancer cells and cancer-associated fibroblasts [34], resulting in increased cell proliferation and migration [35]. Hence, BPA can affect gene transcription through nuclear- and membrane-bound estrogen receptors. On the other hand, it is worth noting that hormones and EDCs, including BPA responses, follow non-monotonic dose–response (NMDR) curves. In the case of BPA, NMDRs have been shown in studies performed on different cell lines such as pituitary, prostate and pancreatic cultured cells, whereas extremely low doses can induce significant effects that are not detectable at higher concentrations, as reviewed in [36]. 

## 4. BPA and Genotoxicity

The genotoxicity evaluation concerning BPA is controversial due to inconsistent data. The human health part of the Scientific Committee on Toxicity, Ecotoxicity and the Environment report under the auspices of the European Commission, concluded, in 2002—in point 2 (Effects assessment), subsection on genotoxicity—that BPA was not mutagenic (Ames test), not genotoxic (standard mouse lymphoma assay), and a non-inductor of gene mutations (Mammalian Cell HPRT Gene Mutation Assay); it affects microtubules and induces aneuploidy in V79 and SHE cells without metabolic activation and it acts as CREST-positive micronuclei in V79 cells [37].

Exposure concentrations equal to or greater than 100 μM of BPA are genotoxic and associated to altered cellular proliferation [38], with concentrations of 50–200 μM directly interfering with cell division and promoting microtubular polymerization, which induces the formation of multipolar spindles in HeLa cells [39]. The induction of micronuclei was also reported in human lymphoblastic cell lines (AHH-1) with a dose range of 54.12–162.8 μM [40] as well as in MCL-5 at 22–132 μM, and further correlated with chromosome non-disjunction at 22–88 μM levels [41]. 

In summary, BPA showed some evidence of genotoxicity activity in vitro, and carcinogenicity studies in animals did not show significant carcinogenic effects; therefore, BPA cannot be considered genotoxic in vivo. The DNA-adducts were not considered to be a concern to humans, since no positive findings for gene mutation and clastogenicity in cultured mammalian cells were observed [37].

Chromosome aberrations studied in somatic and germ cells in vivo did not confirm the clastogenic role of BPA. However, the positive findings in the in vitro gene mutation assays in bacteria and mammalian cells were not confirmed nor ruled out in an appropriate in vivo test on the same genotoxic endpoint. Consequently, a final conclusion on the genotoxic potential of BPA cannot be drawn. It is important to note that the formation of DNA adducts by BPA appears to be identical to the formation of its metabolite glyceraldehyde (IARC Group 2B—possible carcinogenic to humans), based on the induction of local skin tumors in experimental animals after in vivo topical application of BPA to the skin. However, the level of DNA adducts after skin application of BPA is lower than that observed after application of glyceraldehyde, which renders the biological relevance questionable [42].

The study by Naik and Vijayaaxmi [43] showed that BPA did not induce chromosomal aberrations and micronuclei formation in vivo in mice bone marrow; however, it induced achromatic lesions and c-mitotic effects in bone marrow cells. Also, Tsutsui et al. showed BPA induction of numerical chromosomal aberrations and morphological changes in cultured SHE cells [44]. 

Despite these inconsistences, BPA exposure has been shown to cause DNA damage dependently [45] and independently [46] of its estrogenic properties, with its DNA damaging effects being linked indirectly to the generation of reactive oxygen species (ROS). While previous studies have pointed to the DNA damaging effects of BPA, the oxidative-induced DNA damage produced by BPA exposure has not been investigated, nor has BPA exposure in combination with other EDC exposure; or in combination with other DNA damaging agents, especially other oxidizing agents. The ubiquity of BPA exposure, endogenous and exogenous DNA damaging events (oxidative stress and/or environmental toxicants); and the possible combined effect of these exposures can increase the DNA damage load of genomic DNA and have implications for genomic stability and human disease development and progression [46,47]. 

As previously stated, one of the mechanisms suggested to explain BPA genotoxicity is ROS. It remains unclear whether BPA at low (nanomolar) doses can induce ROS and DNA damage in ERα-negative mammary cells [48]. The study from Fic et al., 2013, concluded that BPA induced minor transient DNA damage, measured by comet assay in HepG2 cells at concentrations that are higher than human exposure. Also, the study by Eid et al., 2015, detected DNA damage by comet assay in hepatic tissue, indicating that early life exposure to BPA significantly increased oxidative/nitrosative stress, decreased antioxidant enzyme activities, induced DNA damage and chronic severe inflammation in the hepatic tissue in a time dependent manner [49]. 

The study by Iso et al. [50] reported that BPA induced DNA strand breaks in ER-positive MCF-7 cells and its genotoxicity was dependent, and the study by Tiwari et al. [51] showed increased plasma levels of 8-hydroxydeoxyguanosine, lipid peroxidation, and a decrease in glutathione activity in the liver, suggesting that the oxidative stress could be one of the mechanisms of BPA genotoxicity.

The study by Gassman et al., 2015, supported an immediate effect of BPA in the suppression of the initiation of DNA repair, leading to improved cell survival and a reduction in toxic DNA intermediates [46].

The Pfeifer et al. study examined the effect of low concentrations of BPA on DNA damage, and it concluded that BPA, at environmentally relevant doses, has a genotoxic effect on mammary cells in vitro and induces ROS in these cells [48]. The aforementioned study also showed that BPA at non-cytotoxic levels did induce genotoxic effects—in contrast to the results reported in the study of Audebert et al. [52]—and BPA induced DNA damage markers in cells regardless of the ERα status. Micronuclei formation and structural chromosome aberrations in rat bone marrow as well as DNA damage in lymphocytes was observed in the Tiwari et al. study [51].

It is important to keep in mind that BPA and its genotoxicity still has many “grey” zones, mainly due to the fact that predetermined endpoints used in standard toxicological testing have nothing to do with the modern technological methods that take into account endocrine disrupting compounds and their mode of action; crucial cell-signaling pathways are disrupted and cannot be detected by classical endpoints. Rodent studies reporting low-dose effects of BPA are claimed as irrelevant for the assessment of risks to human health by some groups, because of different toxicokinetics between species. More accurate and sensitive methods are thus required to provide the necessary scientific data required by regulators to assess the safety features and the risk assessment of BPA [53].

## 5. Materials and Methods

In this review, a thorough search was performed for papers available in scientific databases reporting BPA occupational exposure and associated effects. The articles presented and discussed were acquired using several scientific databases such as PubMed and Google Scholar and using the following keywords: bisphenol A (BPA); endocrine disruptor; genotoxicity; occupational exposure; exposure assessment; health effects. Here, we considered experimental studies, epidemiological studies and previous reviews published between 2000–2017. Inclusion and exclusion criteria were established before a bibliographic search. Studies published in languages other than English were considered only if an abstract was available. The articles that, besides written in English, also reported findings regarding BPA occupational exposure and associated health effects, were chosen for further analysis.

## 6. Results

Our results demonstrate that despite the massive BPA production and consumption in European countries, with policarbonate and epoxy resins as the major applications, we have only found 13 studies from 2009 to 2017 performed in the context of occupational exposure in plastic and/or epoxy resin factories in China, 1 in the USA, 1 in Malaysia and 1 in Finland. In the context of thermal paper occupational exposure, we identified three published studies in Brazil, the USA and Italy. These studies are summarized in Table 1.

### 6.1. Occupational Exposure to BPA

#### 6.1.1 Plastic Industry

The plastic industry is one of the most significant and profitable industries worldwide, with increased production every year. 

The European Union Updated European Risk Assessment Report on BPA [69] stated that in Western Europe (2005/2006), BPA production reached 1,150,000 tonnes/year with policarbonate and epoxy resins as the major applications. The European Council of Vinyl Manufacturers (ECVM) reports that at least 1.45 million people work in about 60,000 European PVC resin producing companies that produce 75% of the PVC resin manufactured in Europe [70]. 

Furthermore, currently in Europe, over 280.000 tonnes of epoxy resins are produced every year, whereas the food and drink packaging industry employs 60,000 to 70,000 workers and the processing machinery sector creates about 110,000 jobs [71]. Epoxy-based coating technology utilized in vehicle production processes and presently the automotive industry accounts for 2.3 million jobs. Moreover, the main sector of epoxy applications in Europe is the wind energy industries, using more than 51,400 tonnes of epoxy resins produced by ERC members every year, which together with electronics, account for 69,000 tonnes annually [71]. 

#### 6.1.2. Exposure Assessment

Although the PVC industry is aware of the potentially harmful effects of occupational exposure to some compounds such as 1,2 Dichloroethane (EDC) and Vinyl Chloride monomer (VCM), the potential effects of utilized endocrine disruptors such as BPA, known as the hormone—or endocrine—disruption theory are underestimated and considered safe [70]. In response to the regulatory and media enquiries concerning BPA, the Epoxy Resin Committee has commissioned an independent research agency to conduct studies assessing potential emissions during the life cycle of epoxy resins. The studies analyzed epoxies applications and the greatest plausible sources of possible BPA emissions: water pipes, flooring, marine coatings, automotive, wind rotor blades. However, the report focused on the potential environmental exposures, declaring that epoxies are completely safe once they are processed and applied whereas BPA inhalation was overlooked [72]. These statements are conflicting with the results obtained in a recent study performed in southern Taiwan which demonstrated that, in polycarbonate (PC) molding plants, BPA particle emissions increase during the heating process and that BPA particles are mostly deposited in the nasal cavity (63.37%), alveolus (30.7%), and trachea-bronchus (5.93%). In this plant, airborne BPA concentrations ranged from 32.28 μg/m^3^ to 49.97 μg/m^3^ [73].

Organic extracts from particulate matter of urban outdoor environment air samples demonstrated effective estrogenic effects of BPA associated with enhanced cell proliferation activity observed in a cell-growth assay performed in MCF-7 human breast cancer cells [74]. 

Interestingly, despite the massive BPA production in European countries, the existing occupational exposure studies performed in plastic and/or epoxy resin factories with associated potential occupational diseases arising from BPA exposure have been conducted exclusively in China, whereas in Europe only urine measurements were assessed, in a study from Finland (Table 1). All the performed studies detected BPA levels associated with occupational exposure in the average range of 10 ng/mL to 100 ng/mL. These BPA concentrations are significantly high, particularly considering the BPA levels associated with environmental exposure. The compilation of distinct independent studies revealed that human exposure through diet, estimated on migration values, points to a range of 0.4–4.2 μg/kg body weight/day [2]. However, some inconsistencies have been reported between estimated intake BPA levels and data recorded from biomonitoring studies performed in human blood/serum samples that indicate a steady-state presence of unmetabolized BPA in the range of 0.5–3 ng/mL (2–13 nM) [4,36]. Interestingly, these levels are about 10-fold higher than the worst case predictions for daily human exposure to BPA [22]. Also, physiologically based model studies estimate that, for adult humans, these levels of unmetabolized BPA in blood would require dosages considerably higher than the TDI [75,76] which suggests that either BPA intake is higher than estimated or that it is able to bioaccumulate in the body. Relevantly, in occupationally exposed individuals, the detection rate of serum BPA increases in relation to years of exposure. Higher BPA levels were detected in workers exposed for more than 5 years than in workers exposed for 5 years or less (82.5% vs. 70.1%), and the median BPA concentration was 27.18 ng/mL vs. 9.73 ng/mL, respectively [77]. 

#### 6.1.3. Paper Industry

BPA is also utilized in paper production, comprising development processes of the highest quality thermal paper due to its effectiveness in the manufacture of high-performance products [78]. Contrary to many other applications, in thermal papers, BPA is in its free form (i.e., discrete, non-polymerized). In paper towels made from recycled paper, BPA levels range from 0.55 to 24.1 mg/kg. BPA was also found in 45% paper and cardboard containers used for take-out food reviewed in [4]. In 2015, the European Food Safety Authority (EFSA) indicated that thermal paper is the second biggest source of BPA exposure after the food chain. Some suppliers replaced BPA with its analogue Bisphenol S (BPS), speculatively supposed to be safer [78]. 

The Confederation of European Paper Industries (CEPI) key statistics (2014) reported that the industry structure employed 181,111 workers in 628 companies [79]. 

#### 6.1.4. Exposure Assessment

Considering that BPA is utilized in the production of paper, including thermal paper, workers from thermal paper factories and workers that are exposed in their workplace to paper containing BPA may have increased BPA levels associated with occupational exposure. A recently published study in Finland demonstrated that exposure to BPA is more likely to occur in work tasks that involve the use of pure BPA as workers in thermal paper manufacturing that operate coating machines had median urinary BPA post-shift concentrations in the range of 130–250 μg L^−1^. In this study, considering the detected low air levels of BPA, the authors provided evidence in support of the concern regarding hazardous exposure via dermal contact [66]. A study performed in São Paulo State, Brazil [67] demonstrated that BPA is present in 98% of samples from 190 different thermal receipts, randomly collected, at concentrations ranging from under the quantification limit to 4.3% (mg/100 mg paper). The authors estimated that the daily intake through dermal absorption from handling thermal receipt papers in both environmental and occupationally exposed individuals was median: 1.42 μg/day and 71 μg/day, respectively [67]. Additionally, another study performed in the USA, with the aim of determining whether handling receipt paper results in measurable absorption of BPA via dermal exposure, demonstrated that the mean urine BPA post-shift concentration was significantly higher than in non-cashiers which indicate that thermal receipt paper is a potential source of occupational exposure to BPA [80]. In a recently published study in Italy, the only European county with a BPA assessment of thermal paper, BPA was found in 44 samples at a mean concentration of 107.47 μg/100 mg of paper and its utilized substitute, BPS, was found in 31 samples at a mean concentration of 41.97 μg/100 mg of paper, and both compounds were found in 26 samples. The authors estimated daily intake (EDI) values of BPA and BPS occurring through dermal absorption were 0.0625 μg/day for BPA and 0.0244 μg/day for BPS for the general population, and 66.8 μg/day for BPA and 15.6 μg/day for BPS for occupationally exposed individuals [80]. 

According to the European Chemical Agency (ECHA), the French government has proposed the restriction of BPA use in thermal paper in Europe [81]. However, the European Thermal Paper Association (ETPA) has declared, in an updated statement (2015), the safety of direct thermal papers [82]. The ETPA statement is grounded on reports such as those given by the Zurich University which reported that only negligible quantities of BPA can be absorbed through the skin and thus enters the bloodstream, based on cutaneous penetration migration tests in pig skin [83]. Also, simulation experiments performed by three volunteers from the Finnish Institute of Occupational Health considered that exposure to BPA from handling thermal paper was not relevant [84]. Additionally, the Council for Thermal Paper Facts which is an industry-sponsored informational resource, stated that BPA is safe for workers and for the public. This entity declares that the risk resulting from BPA can be considered insignificant, including for workers with repeated and prolonged contact with cash register receipts [85].

Nevertheless, it is important to note that the referred studies that classify BPA exposure as safe only focused on the cutaneous absorption of BPA through dermal contact; none of them have evaluated the effects caused by the exposure of the detected BPA levels, the potential occupational diseases arising from this type of exposure; they have not evaluated or considered the levels and effects of the combined occupational and environmental BPA exposures. 

The scientific committee of EFSA recommends the benchmark dose (BMD) approach for deriving the reference points for risk assessment and acknowledges this approach as more scientifically accurate than the NOAEL/LOAEL approach [18]. Relevantly, the BMD approach is supported by the REACH legislation guidance document [69].

### 6.2. BPA Occupational Exposure Studies

Despite the magnitude of the plastic and thermal paper industries, reliable data on the number of workers at risk at a European level or the number of occupational diseases arising from BPA exposure is still currently unknown. Remarkably, we did not find any study or report assessing BPA occupational exposure with potentially associated health effects in Europe. Only recently, a study was published reporting occupational exposure to BPA in paint factories, a tractor factory, a composite product factory and a thermal paper factory, located in Finland [66]. 

BPA occupational exposure studies are summarized in Table 1. 

## 7. Occupational BPA Exposure and Human Health Effects

BPA human exposure is in the range of the called “low-doses”, which have been the focus of intense investigation, particularly for the past two decades , reviewed in [11,36,86,87]. BPA “low-dose” effects were defined as biological changes that occur at the concentration range of typical human exposure or lower than the expected NOAEL level of 5000 µg/kg/day, used to establish the oral reference dosage (RfD) [17]. 

For the past years, *in vitro* cell systems have been widely used for the assessment of “low-dose” BPA effects and have evidenced the relevance of these exposures in cellular proliferation and gene expression patterns, reviewed in [88,89]. On the other hand several epidemiological and biomonitoring studies have also been conducted. The data recorded in these studies have allowed to establish positive correlations between urinary or blood BPA concentrations and the prevalence of recurrent human diseases. These include thyroid hormone disruption [90]; reproductive malfunctions [59,91], type-2 diabetes mellitus [92], obesity [93] and pathogenesis of age-related diseases such as coronary and carotid atherosclerosis [94,95,96]. Moreover, several studies have also demonstrated the relevance of exposure to BPA in particular life stages such as embryonic development and early life, characterized as the most vulnerable [97,98] and associated mechanism of action particularly regarding effects in female reproductive system [99] and carcinogenic potential [100]. On the other hand, male susceptibility to xenoestrogens such as BPA is also of particular importance and has been debated in the scientific community focused particularly in sexual dysfunction and infertility [97]. 

However, despite of all the available research regarding BPA effects at environmental relevant levels in humans, for occupationally exposed individuals, which are exposed to higher BPA levels information is still negligible. To the best of our knowledge the studies that evaluated the effects of BPA occupational exposure were performed exclusively in China. 

The studies analyzed in our work have evidenced differences between male and female workers and emphasize the importance of BPA exposure during embryonic development. 

For male workers BPA exposure has been associated with a consistently higher risk of male sexual dysfunction across all areas of male sexual function [9]. BPA exposure has been positively correlated with decreased semen quality and male infertility, for which induction of germ cell apoptosis is considered a primary contributing factor [101]. BPA-induced apoptosis was previously observed in human trophoblastic cells *in vitro* for a concentration of 1 μM [102] as well as in rat ovarian cells in vivo after prolonged exposure [103], indicating a potential negative role in reproductive function. Occupational exposure to BPA resulted in increased urine BPA levels significantly associated with decreased sperm concentration, total sperm count, vitality and motility [59]. Liu and co-workers demonstrated that the presence of elevated BPA urinary levels is also associated with alterations of relevant laboratory parameters that may contribute to male infertility, namely prolactin, estradiol and sex hormone-binding globulin (SHBG) [62]. Increased mean serum BPA concentration has also been associated with decreased mean androstenedione (AD) level (0.18 ng/mL, 95 % CI −0.22 to −0.13) and increased mean serum sex hormone-binding globulin (SHBG) level (2.79 nmol/L, 95 % CI 2.11–3.46) in male workers from exposed to epoxy resin factories [77]. Moreover, in male exposed workers sperm methylation level of long interspersed nucleotide elements (LINE-1), a marker of global genome methylation status, was significantly decreased when compared to unexposed workers [61]. Interestingly, previous reports have demonstrated that BPA exposure in the range detected both in environmental and occupational levels induces transcriptional deregulation of LINE1 in cellular models (HUVEC and HT29) associated with global transcription deregulation [104]. Moreover, the activation of mitogen-activated protein kinases (MAPK) are believed to be involved in the damaging effects of BPA on testicular function associated with disruption of the Sertoli cell tight junction (TJ)-barrier function at the blood-testis barrier. Therefore the use of specific inhibitors against MAPK are being considered a possibility to "manage" the negative health effects and/or "protect" occupationally exposed individuals [105].

For female workers, occupational exposure to BPA was associated with alterations of relevant reproductive hormones including increased prolactin [56,63], progesterone and estradiol levels [63]. 

Alterations in hormone levels, both in male and female workers, are in agreement with the endocrine disruptor activity of BPA, discussed above, and indicate that occupationally exposed workers have an increased risk of endocrine dysfunction associated with BPA exposure. 

Furthermore, authors demonstrated that there are relevant epidemiological evidences that indicate that parental exposure to BPA in the workplace during pregnancy is associated with decreased birth weight of offspring [57] and with shortened anogenital distance in male offspring [58] which substantiates the concern regarding BPA exposure during embryonic development. It is important to notice that considerably high concentrations of BPA have been measured in human placental samples as well as in fetal serum indicating that the placenta does not work as a barrier to BPA and therefore that human developing fetuses are chronically exposed to BPA during all stages of embryonic development [106]. Studies in mice have demonstrated that maternal exposure to BPA can affect the offspring epigenome which resulted in decreased DNA methylation upstream of the *Agouti* gene what was prevented by maternal dietary supplementation with folic acid [107]. Additionally, a genome wide analysis of DNA methylation in the developing mouse forebrain revealed that 64% of the loci analyzed had developmental stage-dependent hyper or hypo methylation alterations associated with BPA exposure [108]. These data indicate that fetal exposure to BPA may affect the fetuses epigenome and consequently gene expression [109]. Altered transcription patterns associated with modifications in DNA methylation have been described for numerous genes in human cells exposed to BPA [110]. 

Prospective studies that correlate early exposure to BPA with health outcomes in children, as well as early markers or predictors of disease later in life, are needed to fully understand the impact of BPA on human health [111].

Overall, these studies clearly indicate that occupational exposure to BPA is an effective risk to the health of the workers and importantly to the health of workers’ descendants. The data collected here have undoubtedly demonstrated that BPA occupational exposure can no longer be overlooked and ignored. 

## 8. Conclusions

Presently, the scientific community debates the epidemiologic/biomonitoring studies and in vitro data of BPA exposure effects with great controversy. It is established, however, that this xegorestrogen promotes variable cellular responses, particularly associated to cell type specificities and critical windows of exposure (e.g., embryonic development and early life) in which humans are more vulnerable. Considering the massive production and consumption of items containing BPA and the elevated number of workers exposed to this xenoestrogen, valuable and effective occupational exposure assessments urgently need to be performed. Importantly, this overview also emphasizes the fact that occupationally exposed individuals are also environmentally exposed to BPA, and therefore evaluation of the aggregate exposures is certainly of the utmost importance in order to conduct a valuable and accurate risk assessment. 

## Figures and Tables

**Table 1:** Summary of Bisphenol A (BPA) occupational exposure studies.

Country	Work Place	*n*	Detected Levels	Health Effects	Reference
China	BPA manufacturers and epoxy resin	167	440–543 μg/g Cr * (urine) 51 μg/m^3^ (Airborne)	-	[10]
China	BPA manufacturers	20	101.94 μg/L (serum)	Semen quality	[54]
China	BPA manufacturers and epoxy resin	123	57.9 μg/g Cr * (urine) 15.31–2.16 μg/m^3^ (Airborne)	Male sexual dysfunction	[9]
China	BPA manufacturers and epoxy resin	427		Male sexual dysfunction	[55]
China	BPA manufacturers	51	-	Prolactin (PRL)	[56]
China	BPA manufacturers and epoxy resin	143	9.1–28.0 μg/g Cr * (urine)	Offspring birth weight	[57]
China	BPA manufacturers and epoxy resin	56	10.8 μg/g Cr * (urine)	Male offspring anogenital distance	[58]
China	BPA manufacturers	218	-	Semen quality	[59]
China	epoxy resin	28	31.96 ± 4.42 μg/g Cr * (urine)	FT3, FT4, TT3, TT4, thyroid-stimulating hormone, glutamic-oxaloacetic transaminase and γ-glutamyl transferase	[60]
China	BPA manufacturers	77	36.23 μg/g Cr * (urine)	LINE-1 methylation	[61]
China	BPA manufacturers and epoxy resin	592	685.9 μg/g Cr * (urine)	Prolactin, estradiol, SHBG	[62]
China	epoxy resin	106	22.2 (μg/g Cr) (urine)	Follicle stimulating hormone, luteinizing hormone, 17β-estradiol, prolactin, and progesterone	[63]
China	epoxy resin	281	18.75 ng/mL (serum)	Androstenedione, SHBG	[61]
USA	BPA manufacturers and epoxy resin	77	0.78–18900 μg/g (urine)	-	[64]
Malaysia	BPA manufacturers		3.81 ng/mL (urine)	-	[65]
Finland	BPA manufacturers		100–170 μg/L (manufacturing liquid paint hardener urine workers) 130–250 μg/L (thermal paper manufacturing urine workers)	-	[66]
Brazil	Thermal paper	-	BQL **—4.3% (mg/100 mg paper)	-	[67]
USA	Thermal paper	77	1.89–2.76 μg/g (urine) 19.3 mg/g (paper)	-	[65]
Italia	Thermal paper	50	107.47 μg/100 mg (paper)	-	[68]

* Creatinine-corrected (mg/g Cr) ** Below the quantification limit.
